# Supplementary material for: Risk-Benefit Assessment of Cereal-Based Foods Consumed by Portuguese Children Aged 6 to 36 Months—A Case Study under the RiskBenefit4EU Project
Source: Nutrients. 2021 Sep 8;13(9):3127. doi: 10.3390/nu13093127 (PMC8467172; doi:10.3390/nu13093127)
Supplement: Supplementary file 1 [file nutrients-13-03127-s001.zip › nutrients-1326388-supplementary.pdf]

Supplementary Materials

Table S1: Summary of the probabilistic approach performed, including the probabilistic distributions assigned to each variable.

Health effect	Model and input parameters	Description
**Nutrition**		
Diabetes Mellitus type 2 (DM2)	$RR_{DM2,fiber} = \exp\left(r_{DM2,fiber} \cdot I_{fiber}\right)$	Dose-response relationship describing change in risk of DM2 due to fiber intake.
	$r_{DM2,fiber} \sim Gaussian(-0.1625, 0.0205)$	Change in risk of DM2 per g fiber/day derived from dose-response RR given by Reynolds et al, 2019 [1]
	$DALY\ rate_{DM2} \sim\ Gaussian(22.42, 5.44)$	Disability-Adjusted Life Years rate for DM2 per 100k, based on the data from GBD 2017 [2]
	$Incidence\ rate_{DM2} \sim\ Gaussian(168.07, 22.44)$	Incidence rate of DM2 per 100k, based on the data from GBD 2017 [2]
Cardiovascular disease (CVD)	$RR_{CVD,fiber} = \exp\left(r_{CVD,fiber} \cdot I_{fiber}\right)$	Dose-response relationship describing change in risk of CVD due to fiber intake.
	$r_{CVD,fiber} \sim Gaussian(-0.2485, 0.0701)$	Change in risk of CVD per g fiber/day derived from dose-response RR given by Reynolds et al, 2019 [1]
	$DALY\ rate_{DM2} \sim\ Gaussian(60.78, 5.94)$	Disability-Adjusted Life Years rate for CVD per 100k, based on the data from GBD 2017 [2]
	$Incidence\ rate_{DM2} \sim\ Gaussian(25.07, 1.94)$	Incidence rate of CVD per 100k, based on the data from GBD 2017 [2]
**Toxicology**		
	0.01 *cases/100,000/year/ng/kg bw/day*	Dose-response relationship for HCC associated with human exposure to aflatoxins [3]
	$DALY\ rate_{HCC} \sim\ Gaussian(9.96, 1.095)$	Disability-Adjusted Life Years rate for HCC per 100k, based on the data from GBD 2017 [2]
**Microbiology**		
Gastrointestinal disease	P = Beta (s+1, n-s+1)	Prevalence of positive cereal boxes in *Bacillus cereus.* With n total number of samples analyzed and s the number of positive, Portuguese data: n_BC=150, s_BC=14, n_IC=35 and s_IC=15.
	$N_{contaminated}$_BC = Cumulative distribution	Level of *Bacillus cereus* in boxes of BC contaminated. Cumulative distribution built on the dataset: 10 samples between 10 and 40 cfu/g (set at 25), 3 at 40 cfu/g and 1 at 180 cfu/g.
	$N_{contaminated}$_IC= Triangular (1, 10, 100)	Level of *Bacillus cereus* in boxes of IC contaminated, in cfu/g.
	N = Bernouilli (P) x $N_{contaminated}$	Estimated level of *Bacillus cereus* in boxes of cereals.
	$I_{B.cereus}$= N x Consump	Daily intake in *Bacillus cereus* in cfu/day, with consumpt the consumption of BC and IC in kg/day.

DR1_B.cereus = 1.0 x 10^3 cfu/g<br>DR2_B.cereus = 5.0 x 10^4 cfu/day	Threshold dose-response expressed as a limit of concentration in cereals (DR1,[4]) or a limit of exposure (DR2, [5]).

Table S2. Median fiber intake classes based on the quartiles of the intake's distribution of the Portuguese children aged between 6 and 35 months and the risk ratios (RR) estimates (95% CI) for type 2 diabetes (DM2) and cardiovascular disease (CVD) for the different scenarios considered.

	**Reference**	**100% BC**	**100% IC**	**Best BC**	**Worst IC**
Fibre (g/day)					
Class 1	5.97	6.12	5.94	7.13	5.66
Class 2	8.13	8.32	8.08	9.91	7.78
Class 3	10.00	10.22	9.93	12.14	9.61
Class 4	12.71	12.99	12.61	15.40	12.28
Risk ratios estimates for DM2					
Class 1	0.886<br>(0.860-0.913)	0.883<br>(0.856-0.911)	0.886<br>(0.860-0.913)	0.862<br>(0.831-0.894)	0.891<br>(0.866-0.917)
Class 2	0.848<br>(0.814-0.883)	0.845<br>(0.810-0.88)	0.849<br>(0.815-0.884)	0.818<br>(0.778-0.859)	0.854<br>(0.821-0.888)
Class 3	0.816<br>(0.776-0.858)	0.813<br>(0.772-0.855)	0.818<br>(0.778-0.859)	0.782<br>(0.735-0.831)	0.823<br>(0.784-0.863)
Class 4	0.773<br>(0.725-0.823)	0.768<br>(0.720-0.82)	0.774<br>(0.727-0.825)	0.732<br>(0.677-0.790)	0.780<br>(0.733-0.829)
Risk ratios estimates for CVD					
Class 1	0.832<br>(0.75-0.920)	0.828<br>(0.744-0.919)	0.833<br>(0.751-0.921)	0.799<br>(0.703-0.903)	0.840<br>(0.761-0.924)
Class 2	0.779<br>(0.676-0.893)	0.774<br>(0.669-0.891)	0.780<br>(0.677-0.894)	0.738<br>(0.62-0.871)	0.787<br>(0.687-0.898)
Class 3	0.736<br>(0.617-0.870)	0.731<br>(0.611-0.868)	0.737<br>(0.619-0.871)	0.690<br>(0.557-0.845)	0.745<br>(0.629-0.875)
Class 4	0.678<br>(0.542-0.838)	0.672<br>(0.534-0.835)	0.680<br>(0.544-0.839)	0.626<br>(0.476-0.807)	0.687<br>(0.553-0.843)

BC: breakfast cereals; IC: infant cereals

1. Reynolds, A.; Mann, J.; Cummings, J.; Winter, N.; Mete, E.; Te Morenga, L. Carbohydrate quality and human health: a series of systematic reviews and meta-analyses. *Lancet* **2019**, *393*, 434–445, doi:10.1016/S0140-6736(18)31809-9.
2. Institute for Health Metrics and Evaluation (IHME) GBD Results Tool 2017 Available online: http://ghdx.healthdata.org/gbd-results-tool.
3. World Health Organization & Joint FAO/WHO Expert Committee on Food Assitives(WHO) *Evaluation of certain contaminants in food: eighty-third report of the Joint FAO/WHOExpert Committee on Food Additives.*; Geneva, 2017;
4. European Food Safety Authority (EFSA) Scientific opionion on the risks for public health related to the presence of Bacillus cereus and other Bacillus spp. including Bacillus thuringiensis in foodstuffs. *EFSA J.* **2016**, *14*, doi:10.2903/j.efsa.2016.4524.
5. Duc, L.H.; Dong, T.C.; Logan, N.A.; Sutherland, A.D.; Taylor, J.; Cutting, S.M. Cases of emesis associated with bacterial contamination of an infant breakfast cereal product. *Int. J. Food Microbiol.* **2005**, *102*, 245–251, doi:10.1016/j.ijfoodmicro.2004.11.022.
